# Australian human and parrot *Chlamydia psittaci* strains cluster within the highly virulent 6BC clade of this important zoonotic pathogen

**DOI:** 10.1038/srep30019

**Published:** 2016-08-04

**Authors:** James Branley, Nathan L. Bachmann, Martina Jelocnik, Garry S. A. Myers, Adam Polkinghorne

**Affiliations:** 1 Department of Microbiology and Infectious Diseases, Nepean Hospital Penrith NSW, Australia; 2 Centre for Animal Health Innovation, University of the Sunshine Coast, Sippy Downs, Australia; 3 iThree Institute, University of Technology Sydney, NSW, Australia

## Abstract

*Chlamydia psittaci* is an avian pathogen and zoonotic agent of atypical pneumonia. The most pathogenic *C. psittaci* strains cluster into the 6BC clade, predicted to have recently emerged globally. Exposure to infected parrots is a risk factor with limited evidence also of an indirect exposure risk. Genome sequencing was performed on six Australian human and a single avian *C. psittaci* strain isolated over a 9 year period. Only one of the five human patients had explicit psittacine contact. Genomics analyses revealed that the Australian *C. psittaci* strains are remarkably similar, clustering tightly within the *C. psittaci* 6BC clade suggested to have been disseminated by South America parrot importation. Molecular clock analysis using the newly sequenced *C. psittaci* genomes predicted the emergence of the 6BC clade occurring approximately 2,000 years ago. These findings reveal the potential for an Australian natural reservoir of *C. psittaci* 6BC strains. These strains can also be isolated from seriously ill patients without explicit psittacine contact. The apparent recent and global spread of *C. psittaci* 6BC strains raises important questions over how this happened. Further studies may reveal whether the dissemination of this important zoonotic pathogen is linked to Australian parrot importation rather than parrots from elsewhere.

*Chlamydiae* are obligate intracellular bacteria that infect a wide variety of anatomical sites in a diverse number of hosts. Four species are known to infect humans including *C. trachomatis, C. pneumoniae, C. abortus* and *C. psittaci*. The latter species is primarily a pathogen of birds where it is responsible for a range of clinical presentations from asymptomatic carriage to severe infection with diarrhoea wasting and death^1^. While this bacterium is an important cause of disease in psittacines and poultry, it can also infect a range of mammals including humans, where it causes the zoonotic illness, psittacosis^1^. In humans, psittacosis is a multi-organ disease presenting with fever, headache, pneumonia and a range of other symptoms that can be mild or result in life-threatening illness^2^. Outbreaks of the disease have been reported through contact with both captive and native birds^3^. Although psittacosis is predominantly acquired through direct bird contact, inadvertent exposure particularly in endemic areas also occurs^4^. Human to human transmission has been reported, raising the spectre of uncontrolled outbreaks^5^.

Australia is home to a large number of non-migratory parrot species. European colonisation in the late 18^th^ century resulted in movement of animals between the old and new worlds. Parrots were moved from Australia to the United Kingdom, Europe and the Americas and urban birds such as pigeons, poultry, sparrows and minor birds were introduced into Australia. Cases of psittacosis have been recognised in Australia since the 1930s^6^,^7^. In the colder or high altitude sites, there have been outbreaks of psittacosis related to wild bird contamination of the environment^3^,^6^. Little is known about the identity of *C. psittaci* strains found in Australian birds, however, or, indeed those that are associated with this suspected transmission via indirect exposure to *C. psittaci*-infected birds.

Globally, the distribution and pathogenicity of *C. psittaci* has been illuminated by molecular typing and comparative studies of a growing number of available *C. psittaci* strains from a variety of hosts^8^,^9^. Perhaps one of the most interesting observations to have come from these comparative genomics studies has been the revelation that the most virulent strains of *C. psittaci*, associated with infections of parrots and zoonotic risk to humans, appear to be clonal^8^,^9^. Strains within this clade (the *C. psittaci* 6BC clade) have been reported in association with epidemics worldwide, highlighting the potential public health impacts of these virulent strains globally. Molecular dating of these strains, performed by Read *et al*.^9^, further suggests that these *C. psittaci* clones emerged recently with a current hypothesis that the importation of South American parrots may be the ultimate source.

As another “new world” source of psittacines, little or nothing is known about the genetic identity of endemic *C. psittaci* strains in Australian humans and parrots and the public health risks for this recognised zoonotic pathogen. In the current study, we have sequenced the largest collection of human *C. psittaci* strains, and a bird isolate from an endemic region of southern New South Wales (NSW), Australia. With one exception, it is thought that all human isolates became infected from indirect contact with *C. psittaci* infected birds. Interestingly, we show that that these isolates cluster within the most pathogenic clade of this zoonotic pathogen described to date, reinforcing the public health risk of direct and indirect exposure to native parrots and raising questions over the ultimate origin of these globally disseminated strains.

## Results

### Genome phylogeny reveals endemic human isolates and an Australian wild parrot *C. psittaci* belong to the pathogenic clonal group

In this study, seven *C. psittaci* strains from an endemic area in southern NSW, Australia^4^,^10^ were sequenced and compared to 19 previously published *C. psittaci* genomes from a range of avian and mammalian hosts. The seven new genomes include six human isolates (Zo-Pa, Fa-An, Fr-Da, Po-An, Ho-Re-upper and Ho-Re-lower) collected from the respiratory tract of five patients with serious clinical symptoms (Table 1). Four out of the five patients were suspected to only have had indirect environmental exposure to wild parrots with one patient (Po-An), keeping psittacines at home. *C. psittaci* Ho-Re-upper and Ho-Re-lower were isolated from the upper and lower respiratory tract, respectively, for the same individual. *C. psittaci* CR009 was isolated from a Crimson Rosella parrot from the same geographic location as the human strains. The seven tissue cultured isolates were sequenced following enrichment of chlamydial DNA using *C. psittaci*-species-specific RNA probes, as previously described^11^. The enriched *C. psittaci* DNA for each isolate was sequenced with a resulting read coverage ranging from 5666X to 156X (Table 2). Following assembly, all strains contained an approximately 1.1 Mbps chromosome and a conserved 8 kb plasmid (Table 2).

**Figure 1:**
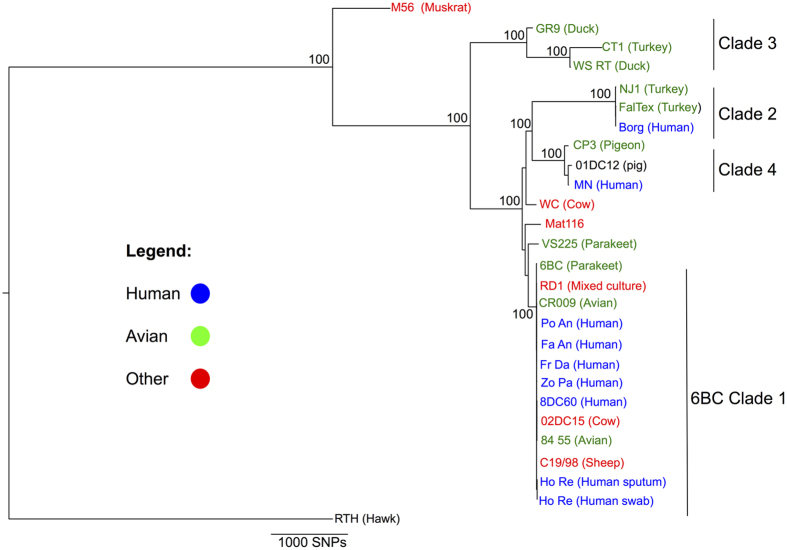
Phylogeny of *C. psittaci*, including newly described Australian parrot and human *C. psittaci* strains isolated from a sympatric area of the Blue Mountains region of NSW, Australia. The maximum-likelihood tree was reconstructed with PhyML with the GTR substitution model based on the 172,540 bp alignments of conserved genomic regions. *C. psittaci* RTH was used as outgroup to root the tree and bootstrap values are shown as percentages. All Australian *C. psittaci* strains cluster in the 6BC clade.

To determine the relationship between the Australian endemic strains and the other *C. psittaci* strains, a phylogenetic tree was constructed based on the alignment of 26 *C. psittaci* genomes. This analysis revealed that all seven of the Australian *C. psittaci* strains cluster within the 6BC clade 1 (Fig. 1), the latter named after *C. psittaci* strain 6BC from a diseased parrot. The 6BC clade 1 has been previously described as a recently emerged pathogenic clonal lineage with very little genetic diversity^9^. The seven Australian strains also have little diversity compared to other strains in this clade with an average of 185 SNPs between the Australian strains and *C. psittaci* 6BC and greater than 81% of these SNPs are found in coding regions. The majority of SNPs are randomly distributed with a single SNP per gene, however there are accumulations of SNPs within genes that encode polymorphic membrane proteins (PMPs), consistent with what was previously described for other strains within this clade^12^. An even smaller degree of variation is observed between the six Australian human *C. psittaci* strains (Zo-Pa, Fa-An, Fr-Da, Po-An, Ho-Re-upper and Ho-Re-lower) and Australian avian *C. psittaci* CR009 with <100 SNPs in total. *C. psittaci* Ho Re (upper) and *C. psittaci* Ho Re (lower) were isolated from the upper respiratory tract and the lower respiratory tract of the same patient, respectively. Since there were no reliable SNP variation detected between the genome sequences of Ho Re (upper) and Ho Re (lower) we concluded that both of these isolates are the same *C. psittaci* strain.

This phylogenetic analysis (Fig. 1) also resolved three additional clades of sequenced *C. psittaci* strains. Clades 2 and 3, also described by Read *et al*.^9^ contain a mixture of *C. psittaci* strains from poultry with one isolated human isolate, Borg. The fourth clade, resolved in our study, consists of *C. psittaci* isolates from humans (MN), a pig (01DC12) and a pigeon (CP3). Compared to the isolates in Clade 1, Clades 2, 3 and 4 have approximately 11013, 7057 and 6389 SNPs between isolates belonging to that clade.

### Recombination between the Australian *C. psittaci* strains and other clades

**Figure 2:**
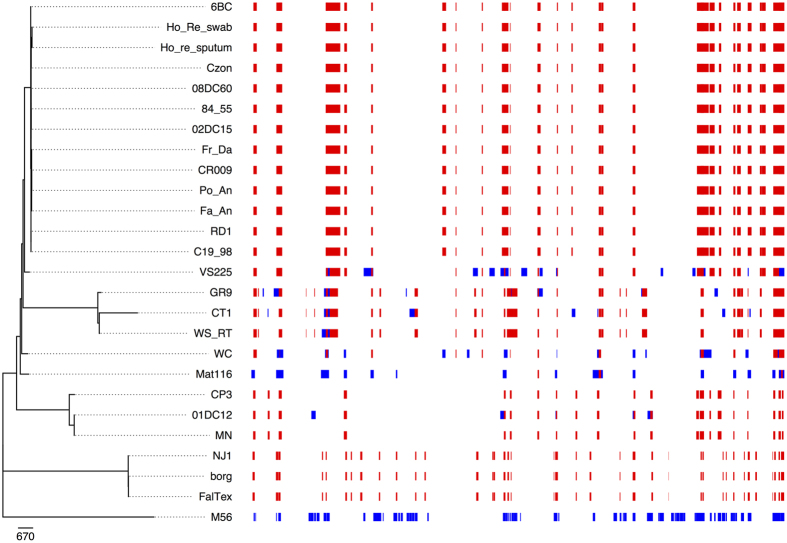
Visual representation of the recombination prediction for all available *C. psittaci* strains including those isolated from Blue Mountains region of NSW, Australia. The phylogeny of *C. psittaci* is shown on the left. For each strain the coloured blocks represent the recombination regions identified by Gubbins. Blue blocks are unique to a single isolate while red blocks are shared by multiple strains through common descent. The horizontal position of the blocks represents their position in the whole genome alignment.

A previous comprehensive genomic analysis of the available set of *C. psittaci* genomes suggested that *C. psittaci* strains undergo extensive recombination^9^. To assess this for the seven Australian *C. psittaci* strains (CR009, Po An, Fa An, Fr Da, Ho Re U, Ho Re L and Zo Pa), their genomes were aligned with 10 complete *C. psittaci* genomes. This produced a whole genome alignment of approximately 1.1 Mbp and recombination segments were predicted with Gubbins 1.4.1 (Fig. 2). All *C. psittaci* strains in the 6BC clade have an identical recombination profile with 16 predicted recombinant regions across the genome including the Australia isolates. The similar recombination profile supports that the 6BC clade is undergoing recent expansion, as none of the strains in this clade have been exposed to recombination since diverging.

### Recalculating the evolutionary timeline for *C. psittaci*

**Figure 3:**
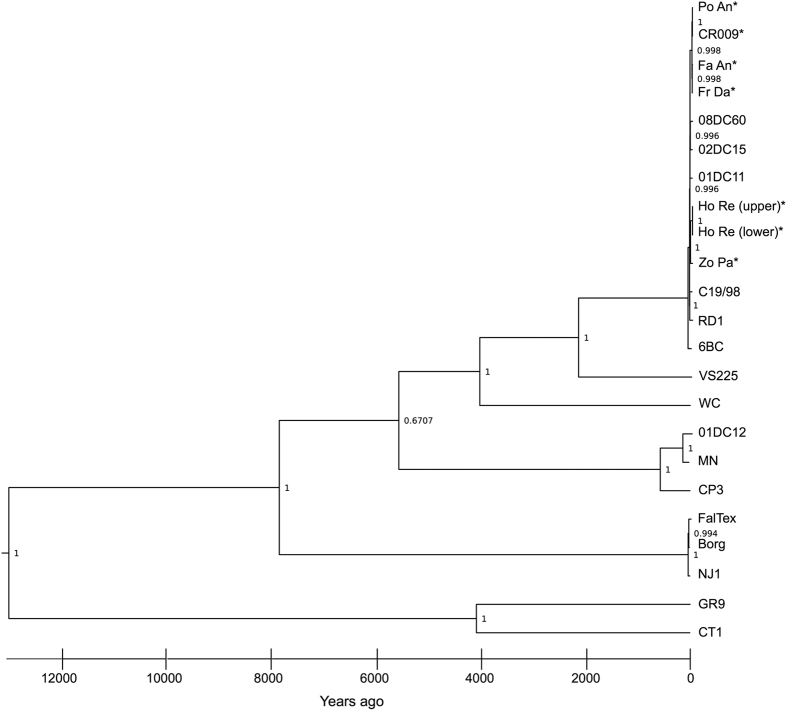
Bayesian phylogenetic reconstruction and predicted evolutionary divergence times of all the available sequenced *C. psittaci* isolates from a variety of hosts from different geographical regions. Bayesian evolution rates and divergence times were predicted using BEAST v2.1.3 under the GTR substitution model using a whole genome alignment of 861,327 bp and with tip dates defined as the year of isolation. Strains with an asterisk (*) were sequenced in this study.

The availability of an additional set of *C. psittaci* strains from a geographically isolated region of the world (i.e. southern Australia), provided an additional opportunity to revisit previous molecular clock predictions for this pathogen^9^. To do so, the substitution rate of *C. psittaci* was predicted using BEAST 2.1.3 and the year of isolation of each strain. Using this data set, the rate of mutation in *C. psittaci* was predicted to be 6.301 × 10^−7^ substitutes per year per site with a 95% credibility interval ranging from 3.394 × 10^−7^ and 8.523 × 10^−7^, which is an accumulation of 0.6 SNPs per year. This is notably slower than previously calculated for this species (1.682 × 10^−4^)^9^. This substitution rate was used to predict the divergence date for each lineage with the most recent common ancestor of all sequenced *C. psittaci* strains calculated to be over 12,000 years ago (Fig. 3). Using this rate, this analysis suggests that the common ancestor of all strains in the 6BC clade emerged approximately 2000 years ago. To test this, we predicted that the strains within the 6BC clade would differ from the parakeet *C. psittaci* VS225 strain by 1393 nucleotide variants. *C. psittaci* VS225 is the closest related strain to the 6BC clade (Fig. 1). Prediction of SNPs between the *C. psittaci* VS225 and the other 6BC clade strains revealed that genomes differ by 2510 nucleotide substitutions. The difference in the SNP prediction from the BEAST analysis and the VarScan prediction is significant as it is larger than the margin of error for these programs^13^. However, this difference is not unexpected, as the increased number of SNPs could be the results of positive selection driving substitution rates in these strains.

## Discussion

Humans are considered an opportunistic host for *C. psittaci;* infections occurring by zoonotic transmission as a result of direct contact with birds^14^. An investigation of an outbreak of psittacosis in the Blue Mountain region of NSW, Australia in 2002, however, found that only 50% of cases were linked to direct contact with birds^3^. This led to psittacosis cases being tracked over a 7-year period from 2003–2009 in the Blue Mountain region, which showed that the causative agent was *C. psittaci* but that direct contact with birds only accounted for a minority of cases^4^. It is hypothesized that there are additional risk factors, such as environmental exposure to bird products that could be responsible for outbreaks of psittacosis.

The unusual nature of endemic psittacosis in the Blue Mountain region raised important questions over the genetic makeup of local *C. psittaci* strains. Six human and a single avian *C. psittaci* isolates collected from the Blue Mountain region were sequenced and compared to other global *C. psittaci* genomes. By performing phylogenetic analyses of human and avian *C. psittaci* strains sequenced in this study, we have shown evidence that human cases within this endemic region are virtually identical and closely related to the one wild parrot isolate. The primary evidence to support this is that all isolates collected over this seven year period, including the isolate *C. psittaci* CR009 from a parrot in the Blue Mountain region, differ by less than 100 SNPs. Furthermore, the identical recombination profile (Fig. 2) of these isolates and the *C. psittaci* genomes in the 6BC clade confirms that the Blue Mountain human and bird *C. psittaci* strains are clonal.

The observation that these endemic Australian isolates all fall within the 6BC clade of *C. psittaci* is of considerable public health importance. While genotyping studies have revealed considerable genetic diversity and epidemiological complexity amongst *C. psittaci* strains globally^8^, the 6BC clade has emerged as the most pathogenic phylogroup described to date. Our study reveals that the Australian human and avian strains cluster tightly within this clonal 6BC clade (Fig. 1). With the exception of the large batch of human isolates described in this study, to date, the majority of strains in this clade have been described from parrots or individuals hypothesized to have been exposed to *C. psittaci*-infected parrots. The founder of this clade, the 6BC isolate was isolated from a parakeet associated with the 1930 worldwide epidemic assumed to have had an origin in South American birds^9^. From a public health perspective, these findings have two important implications: (i) it would appear that *C. psittaci* in Australian psittacines consist of strains from the most virulent clade of this pathogen and (ii) that these strains can be transmitted without direct contact with birds. These findings are timely reminders for veterinarians, medical practitioners and individuals who have direct and, indeed, indirect contact with wild Australian psittacines.

The availability of this additional collection of human and avian *C. psittaci* strains from Australia enabled us to re-test the substitution rates for *C. psittaci* using BEAST. Using the strict clock method, we calculated an overall rate of 6.301 × 10^−7^ substitutes per year per site for this pathogen. This result is significantly different from the calculated substitution rate for *C. psittaci* in Read *et al*. of 1.682 × 10^−4^ substitutions per year per site, determined using the relaxed clock model of BEAST^9^. The strict clock method is the preferred method for analysis of intraspecific data (i.e. samples within a single species while the relaxed clock is used of analysis between different species^15^. Raising further questions over this earlier calculation, it should be noted that the rate calculated with the relaxed clock method is the same as the mutation rates of RNA viruses^16^, while the strict clock calculation rate is more similar to that described for other bacterial species^17^,^18^. With this new rate in mind, dates of the most recent common ancestors of each clade within the known diversity of *C. psittaci* are considerably earlier than previously calculated^9^. This suggests that the date of most recent common ancestor of the 6BC clade is approximately 2000 years ago and that the common ancestor of strains within the 6BC clade itself was approximately 100 years ago.

The global spread of the strains in this recently emerged clade raises questions over its method of dissemination. It was previously hypothesized that the 6BC clade originated from South America parrots and was transported to the USA in the 1930s^9^. The presence of endemic *C. psittaci* strains in Australia belonging to the 6BC clade raises questions over this conclusion, highlighting a potential alternative explanation where Australian parrots may have been the vector for the globally distributed 6BC-like strains. Interestingly, the speculation that Australian parrots may be the source of the global dissemination of pathogenic strains of *C. psittaci* is not a new one. In a review on this subject published 70 years ago, Meyer^19^ discussed that while naturally infected South American parrots were speculated as the source of the global pandemic, this view was not universally held and there was evidence that South American parrot species were not the only sources of infection. Indeed, Meyer^19^ cites one Argentinian review published at the time that speculated “Australian parakeets as the reservoir to which all the evil may be ultimately traced”. An origin for *C. psittaci* in wild Australian birds was also supported by observations by Burnet^6^ that this (i) pathogen was universally distributed amongst all major Australian parrot species; and (ii) it largely resulted in asymptomatic infection, leading to the conclusion that *C. psittaci* had been present in Australia for centuries. Standing against this evidence are folklore stories from South America that speak of a disease of influenza-like disease of man connected to intimate contact with “blue” birds^19^. To date, genetic data on South American *C. psittaci* strains is limited with one study from Argentina suggesting that the majority of human strains present belong to a genetically distinct *C. psittaci* clade (WC) than to the 6BC clade described in this study and elsewhere^20^. Extended studies of the genetic diversity of *C. psittaci* from wild Australian and South American psittacines will obviously be required to resolve this question.

In conclusion, this study reveals that Australian human endemic *C. psittaci* isolates belong the most pathogenic and virulent strain strains of this species. It is suspected that these individuals who all became seriously ill had, at most indirect contact with Australian psittacine, re-emphasizing the zoonotic risk of this often forgotten pathogen and the need for greater public health awareness in this region but also other peri-urban habitats where similar exposure may occur. The factors that influence this indirect zoonotic exposure are still not entirely understood however and more work is required to recognize cases of inapparent psittacosis and the factors that may influence the appearance of cases in endemic regions such as that seen in the Blue Mountains, NSW, Australia. The wide distribution of this pathogenic subclade of *C. psittaci* strains will ensure that such epidemiological investigations are of global public health interest.

## Materials and Methods

### Bacterial strains

The clinical details of each of the human and avian *C. psittaci* isolates analysed in this study can be found in Table 1. Collection of human isolates was previously described as part of clinical investigations^4^. Retrospective analysis of these human isolates was performed in accordance with the National Health and Medical Research Council statement on the Ethical Conduct in Research involving Humans and approved by the Western Sydney Local Health District Human Research Ethics Committee (10/9). The avian *C. psittaci* isolate was collected at necropsy from a dead Crimson Rosella at Mid Mountains Animal Health Centre. Necropsy sampling of this specimen was performed under a New South Wales National Parks and Wildlife Service Permit S11688.

Organisms were cultured in monkey green kidney cell line using two passages in a PC3 laboratory. Extraction of DNA was performed using the NucliSENS easyMAG (BioMerieux), according to the manufacturer’s instructions.

### Targeted enrichment and genome sequencing

The total amount of extracted DNA for the seven *C. psittaci* isolates were used as the template for probe hybridization targeted enrichment. The 120-mer RNA probes were designed at the Institute for Genome Sciences (IGS), with the assistance of Agilent Technologies, spanning across the forward strand of the *C. psittaci* 6BC genome (accession number: CP002586). Agilent Technologies synthesized the custom-designed *C. psittaci* probe set. The targeted enrichment and library construction was done using the *C. psittaci* specific probes and a SureSelectXT reagent kit. The resulting seven *C. psittaci* libraries were sequenced on a single channel of an Illumina HiSeq instrument (101 bp paired-end reads). Read quality was checked with FASTQC; read filtering and trimming was performed with Trimmomatic version 0.32^21^. Genomes were assembled using Spades 3.0.0 with k-mer values of 15, 21, 33, 51 and 71^22^. Plasmid contigs were identified by using BLAST to align all assembled contigs against a database of *C. psittaci* plasmid sequences collected from the National Center for Biotechnology Information (NCBI). The sequence data was submitted to the NCBI short read archive (SRA) database with the accession numbers listed in Table 2.

### Phylogenetic and recombination analyses

A core genome alignment using the seven newly sequenced *C. psittaci* genomes (Table 1) and 20 publically available *C. psittaci* genomes (Supplementary Table 1) were performed using Mugsy 1.2.3 with default settings^23^. A custom Perl script was used to concatenate conserved regions that align across all 27 genomes into a single alignment. Poorly aligned regions from the concatenated alignment were removed using GBLOCKS version 0.91b with the minimum length of a block set to 5, and no gap positions allowed, to produce a final core alignment of 172,540 bp. A phylogenetic tree was then constructed from the core alignment with PhyML 3.1^24^ using the generalized time reversible (GTR) model. Bootstrap values were calculated using 500 replicates. The *C. psittaci* RTH strain was used as an outgroup to root the tree. Recombination analysis was performed on the final core genome alignment (excluding C. psittaci RTH) using Gubbins v1.4.1 on default settings^25^.

### Evolutionary Analysis

For the Bayesian analysis of substitution rates and divergence time, a subset of 16 publically available *C. psittaci* genomes with reliable isolation dates (*C. psittaci* 08DC60, *C. psittaci* 02DC15, *C. psittaci* 01DC11, *C. psittaci* C19/98, *C. psittaci* RD1, *C. psittaci* 6BC, *C. psittaci* VS225, *C. psittaci* WC, *C. psittaci* 01DC12, *C. psittaci* MN, *C. psittaci* CP3, *C. psittaci* FalTex, *C. psittaci* Borg, *C. psittaci* NJ1, *C. psittaci* GR9 and *C. psittaci* CT1) were aligned against the genome of the seven Australian *C. psittaci* strains with Mugsy 1.2.3^23^. *C. psittaci* RTH was excluded because it was too divergent from other *C. psittaci*; it has been suggested that this strain belongs to a separate species^26^. The core genome alignment was processed and filtered using the same method as described for the phylogenetic analysis. The substitution rate of *C. psittaci* was predicted using BEAST 2.1.3^27^ with the GTR substitution model and tip dates as the year of isolation. The analysis used 145 million Markov chain Monte Carlo (MCMC) iterations sampled every 1,000 generation with the first 10% discarded as burn-in. The evolutionary analysis used the strict clock model and the Coalescent Bayesian skyline model built into BEAST. Tracer 1.5 was used to analysis the BEAST results and TreeAnnotator 2.1.2 was used to draw the divergence timeline of *C. psittaci*.

### SNP analysis

The identification of SNPs in strains that belong to the 6BC clade was predicted using Mauve snapshot 2015-02-25^28^ to align each of the 6BC clade strains against *C. psittaci* VS225. Mauve was used to predict SNPs as some of the public *C. psittaci* strains didn’t have read data available. *C. psittaci* VS225 was used as reference as it is the closest related strain to the 6BC clade, as determined by our phylogenetic analysis.

## Additional Information

**How to cite this article**: Branley, J. *et al*. Australian human and parrot *Chlamydia psittaci* strains cluster within the highly virulent 6BC clade of this important zoonotic pathogen. *Sci. Rep.* **6**, 30019; doi: 10.1038/srep30019 (2016).

## Supplementary Material

Supplementary Information

## Figures and Tables

**Table 1:** *C. psittaci* strains sequenced as part of this study.

Isolate name	Host species	Sample	Year of isolation	Geographic location	Patient age	Sex	Signs/lx	Explicit Bird contact
Fr Da	Human	Throat swab	2003	Wentworth Falls, NSW	58	M	Pneumonia, hepatitis	N
Zo Pa	Human	Throat swab	2008	Katoomba, NSW	47	M	Pneumonia, hepatitis	N
Fa An	Human	Throat swab	2009	Lawson, NSW	80	M	Hyponatraemia, Atrial fibrillation	N
Ho Re upper	Human	Throat swab	2012	Oberon, NSW	68	M	—	N
Ho Re lower	Human	Bronchial aspirate	—	—	—	—	—	—
Po An	Human	Throat swab	2009	Springwood, NSW	45	M	Pneumonia, hepatitis	Y; psittacines at home
CR009	Crimson Rosella (*Platycercus elegans*)	—	2006	Wentworth Falls, NSW			Wasting	NA

**Table 2:** *C. psittaci* genome sequence statistics.

Strain	Percentage of chlamydial reads (%)	Number of chamydial contigs	Largest Contigs	Mean read depth	Standard deviation of read depth	Genbank accession number
CR009	85	11	776 990	3818	1064	LZRX01000000
Zo Pa	85	8	777 116	5666	928	LZRY01000000
Fa An	12	57	475 928	252	95	LZRZ01000000
Fr Da	31	8	778 122	156	68	LZSA01000000
Ho Re upper	83	7	777 133	1616	346	LZRE01000000
Ho Re lower	83	78	777 133	1798	377	LZRF01000000
Po An	13	13	778 148	283	96	LZRG01000000
