# Supplementary material for: Australian human and parrot Chlamydia psittaci strains cluster within the highly virulent 6BC clade of this important zoonotic pathogen
Source: Sci Rep. 2016 Aug 4;6:30019. doi: 10.1038/srep30019 (PMC4973220; doi:10.1038/srep30019)
Supplement: Supplementary Information [file srep30019-s1.doc]

**Supplementary information for:**

**Australian human and parrot *Chlamydia psittaci* strains cluster within the highly virulent 6BC clade of this important zoonotic pathogen**

James Branley^1#^, Nathan L. Bachmann^2#^, Martina Jelocnik^2^, Garry S.A. Myers^3^, Adam Polkinghorne^2*^.

| **Supplementary Table 1**: Public *Chlamydia psittaci* genomes used in this study | | | |
| --- | --- | --- | --- |
|  |  |  |  |
| **Strain** | **Isolation year** | **Accession Number** |  |
| GR9 | 1960 | CP003791 |  |
| VS225 | 1991 | CP003793 |  |
| 02DC15 | 2002 | CP002806 |  |
| 84 55 | - | CP003790 |  |
| FalTex | 1980 | SRR652457 |  |
| CT1 | 1954 | SRR652462 |  |
| MN | 1936 | SRR652461 |  |
| 6BC | 1983 | CP002586 |  |
| CP3 | 1958 | SRR652460 |  |
| 01DC11 | 2001 | CP002805 |  |
| NJ1 | 1954 | CP003798 |  |
| RD1 | 2010 | FQ482149 |  |
| C19 98 | 1998 | CP002804 |  |
| Mat116 | - | CP002744 |  |
| WC | 1963 | CP003796 |  |
| 08DC60 | 2008 | CP002807 |  |
| WS RT | - | CP003794 |  |
| M56 | 1961 | CP003795 |  |
| 01DC12 | 2001 | HF545614 |  |
| borg | 1944 | SRR652470 |  |
| RTH | 2003 | SRR652473 |  |
